# Time moderates the interplay between 5-HTTLPR and stress on depression risk: gene x environment interaction as a dynamic process

**DOI:** 10.1038/s41398-022-02035-4

**Published:** 2022-07-11

**Authors:** Claudia Delli Colli, Marta Borgi, Silvia Poggini, Flavia Chiarotti, Francesca Cirulli, Brenda W. J. H. Penninx, Francesco Benedetti, Benedetta Vai, Igor Branchi

**Affiliations:** 1 Center for Behavioral Sciences and Mental Health, Istituto Superiore di Sanità, Rome, Italy; 2 PhD program in Pharmacology and Toxicology, Department of Physiology and Pharmacology “Vittorio Erspamer”, Sapienza University of Rome, Rome, Italy; 3 Department of Psychiatry, Amsterdam UMC, Vrije Universiteit, Amsterdam, The Netherlands; 4 Psychiatry & Clinical Psychobiology, Division of Neuroscience, IRCCS San Raffaele Scientific Institute, Milan, Italy; 5 Università Vita-Salute San Raffaele, Milan, Italy

**Keywords:** Depression, Predictive markers

## Abstract

The serotonin-transporter-linked promoter region (5-HTTLPR) has been widely investigated as contributing to depression vulnerability. Nevertheless, empirical research provides wide contrasting findings regarding its involvement in the etiopathogenesis of the disorder. Our hypothesis was that such discrepancy can be explained considering time as moderating factor. We explored this hypothesis, exploiting a meta analytic approach. We searched PubMed, PsychoINFO, Scopus and EMBASE databases and 1096 studies were identified and screened, resulting in 22 studies to be included in the meta-analyses. The effect of the 5-HTTLPR x stress interaction on depression risk was found to be moderated by the following temporal factors: the duration of stress (i.e. chronic vs. acute) and the time interval between end of stress and assessment of depression (i.e. within 1 year vs. more than 1 year). When stratifying for the duration of stress, the effect of the 5-HTTLPR x stress interaction emerged only in the case of chronic stress, with a significant subgroup difference (*p* = 0.004). The stratification according to time interval revealed a significant interaction only for intervals within 1 year, though no difference between subgroups was found. The critical role of time interval clearly emerged when considering only chronic stress: a significant effect of the 5-HTTLPR and stress interaction was confirmed exclusively within 1 year and a significant subgroup difference was found (*p* = 0.01). These results show that the 5-HTTLPR x stress interaction is a dynamic process, producing different effects at different time points, and indirectly confirm that s-allele carriers are both at higher risk and more capable to recover from depression. Overall, these findings expand the current view of the interplay between 5-HTTLPR and stress adding the temporal dimension, that results in a three-way interaction: gene x environment x time.

## Introduction

Stress represents one of the most relevant risk factors for psychopathologies, including major depression [1, 2]. However, vulnerability to stress differs among individuals and its consequences are not predictable just by taking into account the magnitude of the stressor. While serious life-threatening stressful events do not affect some individuals, milder stressors may trigger depression in others [3].

Among the factors that potentially explain inter-individual differences in the vulnerability to stress, gene x environment interactions, where different alleles of a polymorphism moderate the effect of the environment on the individual, play a key role [4]. One of the most investigated polymorphisms concerns the promoter region of the serotonin transporter gene (5-HTTLPR). The short (s) allele is associated with a reduced transcription level of the serotonin transporter compared with the long (l) allele [5, 6]. The action of the 5-HTTLPR in interaction with the environment has been described for the first time by Caspi and collaborators [7]. They reported that individuals carrying either one or two copies of the s allele are more likely to develop major depressive disorder in response to stress than individuals homozygous for the l allele. Since then, many studies have confirmed these findings [8–11]. However, many others reported no evidence of such interaction [12–14]. Meta-analyses, also, have come to discordant conclusions [15–19], proposing various reasons to reconcile these discrepancies including differences in study design and in the methodologies used in the assessment of psychopathology. Recently, the view of 5-HTTLPR as producing no relevant effects is gaining momentum. One of the most recent and largest meta-analyses on this polymorphism, exploiting different strategies to subgroup individuals or variables, found no statistically significant interaction between the 5-HTTLPR and stress [20].

The potential risk action of the 5-HTTLPR is classically interpreted according to the diathesis-stress model which posits that a specific allele is associated with high vulnerability. More recently a different model—the differential susceptibility to environment—has proposed that individuals bearing the different alleles of the polymorphism do not differ in terms of vulnerability, but in terms of plasticity, i.e. the susceptibility to change behavioral outcome [21–23]. This is an important conceptual shift toward a novel theoretical framework: from viewing the two alleles as associated to different traits of vulnerability (i.e., vulnerable or not vulnerable), to considering the polymorphism as a regulator of a dynamic process (i.e., more or less plasticity). Accordingly, here we hypothesized that the role of the 5-HTTLPR x stress interaction clearly emerges when assessing its effects from a dynamic process perspective. Given the critical role of time in defining dynamic processes, we expected that temporal factors, such as duration of stress and the length of the time interval between end of stress and assessment of depression are key in determining the outcome of the 5-HTTLPR x stress interaction. In particular, interpreting the different levels of plasticity associated to the two alleles as different rates of change in brain function and behavioral outcome, we expect that (i) the different risk of depression in s- and l-carriers emerges with time and is thus evident only following chronic stress. In addition, (ii) since s-carriers are more plastic than l-carriers, they are both at higher risk and more capable to recover. Therefore, s-carriers show increased risk to depression only at short time intervals and the 5-HTTLPR x stress interaction is no more significant following long-time intervals. Differences in the risk of depression when stratifying studies for duration of stress (i.e. chronic vs. acute) and time interval between the end of stress and assessment of depression (i.e. within 1 year vs. more than 1 year) are expected to confirm our hypothesis.

## Methods

### Overview

This meta-analysis seeks to clarify the effect of the interaction between 5-HTTLPR and stress on depression (i.e., diagnosis or depressive symptoms) and how time moderates such interplay. This review was registered with PROSPERO (registration number: CRD42021286237) and was reported according to the Preferred Reporting Items for Systematic reviews and Meta Analyses statement (PRISMA) reporting guidelines (Supplementary Methods 1).

### Search strategy and selection criteria

A systematic literature search was conducted in four online databases (PubMed, PsychoINFO, Scopus and EMBASE) from their inception to July 22th, 2020. The search strategy consisted of three main components: stress (e.g., trauma* OR stress* OR adverse OR life event*), genetic polymorphism of serotonin (e.g., 5-HTTLPR OR serotonin transporter gene polymorphism OR serotonin transporter gene polymorphic region), and depression (e.g., depress* OR psychological distress OR mental illness* OR mood disorder*). The complete search strategy is presented in Supplementary Methods 2. Reference lists of relevant identified systematic reviews were searched for additional eligible papers.

After the removal of duplicates, three authors (MB, SP, and CDC) independently screened article titles and abstracts according to the eligibility criteria. Studies were retained for the next stage of screening (full-text analysis) and disagreements were resolved through discussion and by consulting an additional investigator (IB).

We excluded studies that were not original research (e.g., reviews, editorials, commentaries) and/or no full-text articles (e.g., meeting abstracts); studies written in languages other than English; studies where the 5-HTTLPR variant was not genotyped; studies in which the subjects were not exposed to stress; studies that did not provide measurements of depression (including symptoms and/or diagnosis); studies that did not report the effect of 5-HTTLPR by environment interaction; studies that did not include information on the time between stress exposure and assessment of psychopathology; studies that reported the onset of depression during the pregnancy or 4 weeks after delivery (e.g., postpartum depression); studies that did not report association measures [Odds Ratio (OR) or Logistic regression coefficient (β)].

We included studies that: (1) assessed the effect of the interaction between 5-HTTLPR and stress on depression diagnosis and/or symptoms; (2) provided information about the time interval between stress and depression assessment; (3) reported association measures (OR or β).

When two or more studies included the same population and reported an overlapping sample, the study with the smallest dataset was excluded from the meta-analysis.

Rayyan QCRI [24] was used for the screening process.

### Outcome

The outcome was the incidence of depression (i.e., depressive episodes and/or depressive symptoms) in both clinical and general populations. Risks were assessed through a combination of ORs and adjusted ORs (aORs) and associated 95% confidence intervals (95% CIs). For the outcome, we considered the effect of the following variables: type of stress (acute [e.g., occasional stressful events] vs. chronic stress [e.g., childhood maltreatment, family-related stress]) and time interval (i.e., time between stress and assessment of depression). In addition, in sensitivity analyses, we examined whether the tool used for the assessment of depression (i.e., clinician-observer scales and self-reported scales) impacted our findings (Supplementary Table 1).

### Data extraction and management

For each study, two authors (SP, CDC) independently extracted the following data: first author’s surname, year published, country, sampling (e.g., name of the cohort), study design, number of participants, female percentage, type of stress (e.g. childhood maltreatment, family-related stress, stressful life events), tools used for stress assessment, age (mean and/or range) at which the depression was assessed, tool used for the assessment of depression (Supplementary Table 1), crude OR and/or aOR and their 95% CIs_OR_, covariates included in the model for aOR, β and related standard error (SE_β_).

For each study, three authors (MB, SP, CDC) calculated the time interval (that is, the time interval between stress and depression) in the following way. In the case of acute adverse conditions, the time interval was defined as the maximum period of time within which the stress might have occurred (e.g.,events occurred within the year preceding the assessment of depression have a time interval of 1 year). In the case of chronic stress, the time interval was defined as the period of time between the end of the chronic stress (i.e., 18 years of age in the case of family adversity and childhood maltreatment) and the assessment of depression (mean age at which the assessment was carried out). If the depression assessment overlapped with the stress period (e.g., the effect of family-related stress on adolescent depression) or the stress represented a permanent condition (e.g., discrimination or low socio-economic status) the time interval was considered as zero.

### Quality assessment

The risk of bias related to study quality was carried out by using the critical appraisal tools of Joanna Briggs Institute (JBI) [25]. This tool designed different checklists of items for different studies design (i.e., longitudinal, cross-sectional, and case-control) and is recommended by the Cochrane Methods [26]. The available answers for each item were “yes”, “no”, “not-applicable” and “unclear”. The risk of bias of individual studies was determined with the following cutoffs: low risk of bias if 70% of answers scored yes, moderate risk if 50 to 69% questions scored yes, and high risk of bias if yes scores were below 50% [25, 27]. Quality assessment was done by four authors (FC, MB, SP, CDC) and any disagreements were resolved by discussion.

### Statistical analysis

All statistical analyses have been performed using the R program (version 4.0.5) and *meta* package [28]. For both main and subgroup analyses, the effect size measures of the risk of depression were crude ORs and aORs related to the gene x environment interaction pooled together; findings were presented in forest plots.

When studies reported β and related SE_β_, the former was transformed into OR and the latter was used to calculate the 95% CIs_OR_ (Supplementary Methods 3a). Since the R function (*metagen)* used to perform the meta-analyses requires information on logSE_OR_ and logOR as inputs, all ORs and related standard error (SE_OR_) (Supplementary Methods 3b) were transformed by applying the R function *log*.

To model between-study variance we applied the DerSimonian and Laird random-effects model, a conservative approach when heterogeneity among the studies cannot be excluded. The heterogeneity among the results was explored by using Cochran’s Q and I^2^ statistics; the heterogeneity was categorized as low (*I*^2^ = 25%), moderate (*I*^2^ = 50%), and high (*I*^2^ = 75%) [29].

Heterogeneity was investigated by means of subgroup analyses. Studies were split into subgroups based on duration of stress (chronic vs. acute), time interval between the end of the stress and the assessment of depression (longer than 1 year vs. shorter than or equal to 1 year) and types of tool used for the assessment of depression (clinician-observer scales vs. self-reported scales). We selected the time interval of 1 year because, on the one hand, several authors proposed intervals up to 1 year as periods in which the gene x environment effects are strongest [30–33] and, on the other, this interval was compatible with the time intervals analyzed in the included studies.

Subgroup difference tests were performed to determine whether the effect of the 5-HTTLPR interaction varies significantly among subgroups of studies defined by temporal factors as duration of stress and time interval between end of stress and assessment of depression [34].

To assess the robustness of our results, a series of sensitivity analyses were performed by repeating the main analysis substituting alternative decisions that were arbitrary: (i) when investigating the moderating effect of the duration of the stress, if any of the studies included in this analysis reported independent effects for both chronic and acute stress, the effect of the stress closer in time to the assessment of depression was considered; (ii) when investigating the moderating effect of the time interval, if the same study reported independent stress events at different time intervals, the shortest interval was considered; (iii) when investigating the moderating effect of the diagnostic tool, if the same study assessed depression with different tools, the tool categorized as clinician-observer scale was considered.

To assess potential publication bias, a funnel plot of study effect sizes against standard errors was visually inspected for asymmetry. Asymmetry was also statistically tested with Egger’s bias test with *p* < 0.05 indicating asymmetry.

## Results

**Fig. 1:**
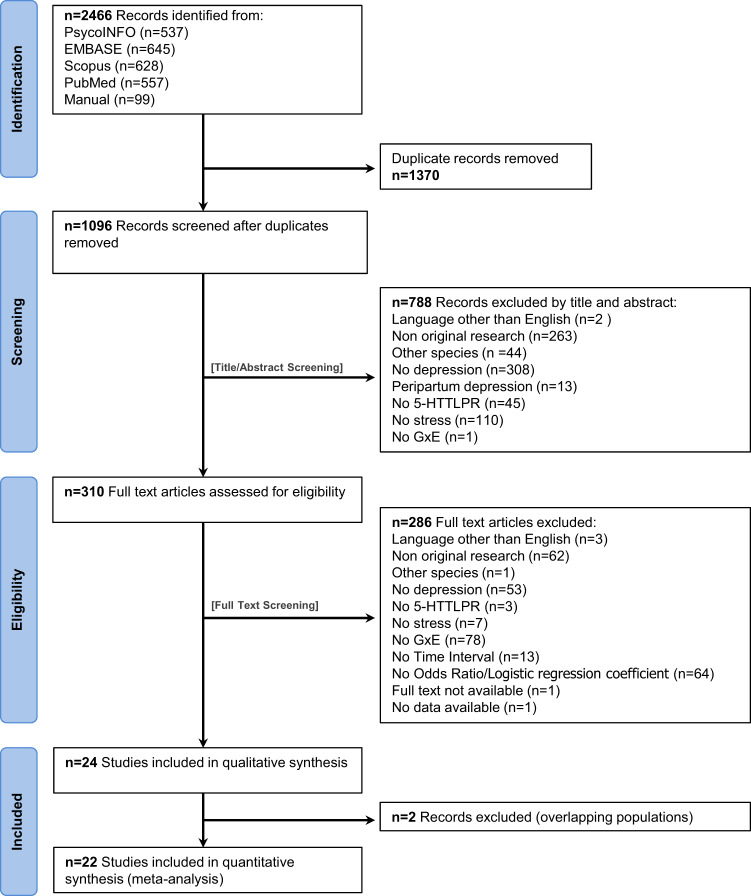
PRISMA flow diagram. Diagram of the literature search (identification) and selection process (screening, eligibility, inclusion).

Our search identified 2466 publications from inception to 2021. After exclusion of duplicates, 1096 records were screened, resulting in 310 publications for eligibility assessment. Following full-text reading, 24 studies met the inclusion criteria for the meta-analysis (Fig. 1). As three studies [35–37] considered overlapping populations, we excluded from the analyses those with the smallest samples (Supplementary Table 2). Characteristics of the 22 articles included in the meta-analysis [7, 14, 37–56] are summarized in Table 1.

**Table 1:** Characteristics of the studies included in the meta-analysis.

Study	Sampling	Country	Study Design	*n*	Sex (% F)	Stressor	Age of depressive assessment [mean (sd), range]	Depression tool^1^	Stress tool
Caspi, 2003a	All births in Dunedin	New Zealand	Longitudinal	847	48%	Stressful life events between the ages of 21 and 26	26	DIS	Life-history calendar
Caspi, 2003b*						Childhood maltreatment			
Chipman, 2007a	Community survey of people aged 20–24 in Canberra (PATH study)	Australia	Cross-sectional	2095	52,10%	Stressful life events over 6 months	20–24	GDA-S	LTE
Chipman, 2007b						Childhood adversity up to the age 16			17-item list of adversities
Chipman, 2007c	Children born in the Australian state of Victoria between September 1982 and January 1983 (ATP study)	Australia	Longitudinal	584	50,60%	Number of family stressor over the previous 12 months	15–16	SMFQ	6-item index consisting of unemployed father, father in unskilled occupation, many family moves, large family size, non-intact family, and high levels of family stress in the previous 12 months
Chipman, 2007d						Persistent family adversity over a 6-year period			
Chipman, 2007e				544	51,50%	Number of family stressor over the previous 12 months	17–18		
Chipman, 2007f						Persistent family adversity over a 6-year period			
Coventry, 2010a	Australian NHMRC Twin Register	Australia	Longitudinal	3243	64,10%	Stressful life events in the preceding 12 months	32.3 (13.6), 18–95	SSAGA	HLQ adapted from the LTE
Coventry, 2010b					65,90%			HLQ	
Cutuli, 2013a	Minnesota Longitudinal Study of Risk and	USA	Longitudinal	157	51,60%	Childhood maltreatment	8	CDR-S	Direct observation, caregiver interviews, reviews of child protection and medical records when available, and teacher interviews
Cutuli, 2013b							8-17.5	K-SADS	
Cutuli, 2013c**							17.5-28	SCID	
Eley, 2004	GENESIS study	UK	Cross-sectional	369	58,50%	Family environmental risk	dic-19	SMFQ	SPQ; LTE; Parental educational level
Fandin˜o-Losada, 2013a	Longitudinal study of mental health among person living in Stockholm Country (PART)	Sweden	Longitudinal	1758	59,70%	Parental separation before 18 years old	44.7 (12.3)	MDI	Questionnaires contained questions on death of parent and divorce/separation of the parents
Fandin˜o-Losada, 2013b						Partner separation during the past 12 months			Questionnaires contained questions on the occurrence of death of partner and divorce/separation from partner
Gilliespie, 2005	Australian National Health and Medical Research Council Twin Register (ATR)	Australia	Cross-sectional	1091	67,80%	Stressful life events during the past 12 months	39 (11), 19–78	SSAGA; SCL-90	LTE
Gutierrez, 2015	PREDICT-gene study between October 2005 and February 2006	Spain	Longitudinal	2679	69,70%	Childhood maltreatment	50.33 (14.91); 18–75	CIDI	CTQ
Haberstick, 2016a	Wave III of National Longitudinal Study of Adolescent to Adult Health (Add Health)	USA	Longitudinal	4724	51,10%	Childhood maltreatment prior to age 12	22–26	CES-D	21-item scale occurring in five domains five domains of experiences: health, housing,employment, finance, and relationships
Haberstick, 2016b**						Stressful life events			21- items to create stressful life events scale
Hankin, 2015	University of Denver and Rutgers University (GEM study)	USA	Longitudinal	665	55%	Chronic stress in peer relationship	11.6 (2.4); 7–16	K-SADS	YLSI
Juhasz, 2015a	NewMood study	Hungary/UK	Cross-sectional	2358	69%	Recent negative life events	32.79 (0.22), 18–60	SCID	LTE
Juhasz, 2015b						Childhood trauma			CTQ
Juhasz, 2015c						Recent negative life events		BSI-DEP	LTE
Juhasz, 2015d**						Childhood trauma			CTQ
Kim, 2017	Department of Cardiology of Chonnam National University Hospital (CNUH)	Sud Korea	Longitudinal	1152	n.a	Stressul life events during	n.a	MINI	LTE
						the 3 months preceding the Acute Coronary Syndrome			
Kudinova, 2015	n.a	n.a	Case-control	355	100%	Childood trauma	40.11 (6.79)	SCID	CTQ
Laucht, 2009a	Mannheim Study of Children at Risk	Germany	Cross-sectional	309	54%	Family adversity	19	BDI	Enriched Index
Laucht, 2009b						Stressful life events between 15 and 19 year			MEL
Laucht, 2009c**						Family adversity	15–19	SCID	Enriched Index
Laucht, 2009d**						Stressful life events between 15 and 19 year			MEL
Özçürümez, 2019	Başkent University Faculty of Medicine	Turkey	Case-control	137	78,80%	Childhood maltreatment	37.76 (8.46)	CIDI; BDI	CTQ
Power, 2010a	Electoral rolls between 1999 and 2001 in Montpellier	France	Longitudinal	1421	58,80%	Stressful life events within 12 months	>65	MINI	Structured questionnaire
Power, 2010b								CES-D	
Quinn, 2012	Brain Resource International Database	Australia	Case-control	240	59,30%	Early life stress	37.89 (13.38)	MINI; HARSD	ELSQ
Rocha, 2015	1993 Pelotas Birth Cohort Study	Brazil	Longitudinal	2392	54,30%	Childhood maltreatment	18–19	MINI	7- retrospective questions
Roy, 2011	New Jersey Medical School	USA	Cross-sectional	150	57%	Childhood maltreatment	29.5 (10.1)	BDI	CTQ
Sales, 2015	Health clinics and country health department from July 2005 and June 2007	USA	Cross-sectional	304	100%	Racial discrimination during the past year	18.09 (1.40); 14–20	CES-D	13-items version of SRE
Surtees, 2006a	General practice registers (EPIC Norfolk 1993–1997)	UK	Cross-sectional	4175	46,70%	Number of adult events or difficulties within 1 years	60.3 (9.1); 41–80	HLEQ	LTE
Surtees, 2006b						Number of adult events or difficulties within 5 years			
Surtees, 2005c						Adverse experiences in childhood (0–16 years)			HLEQ
Wilhelm, 2006a	Postgraduate teachers course in 1978	Australia	Longitudinal	127	66,90%	Personal life events within over 1 year	47.7 (2.8)	DIS/CIDI	Self-report questionnaires
Wilhelm, 2006b						Personal life events within over 5 year			

^1^ Supplementary Table S1

Subsets of data belonging to the same study are reported in different lines (indicated by letters). LTE List of Threatening Events, SPQ Social Problems Questionnaire, CTQ Childhood Trauma Questionnaire, YLSI Youth Life Stress Interview, MEL Munich Events List, ELSQ Early Life Stress Questionnaire, SRE Schedule for racist events, HLEQ Health and Life Experiences Questionnaire.

*not report association measures (not included in the meta-analyses)

**no time interval (not included in the meta-analyses)

### Overall effect

**Fig. 2:**
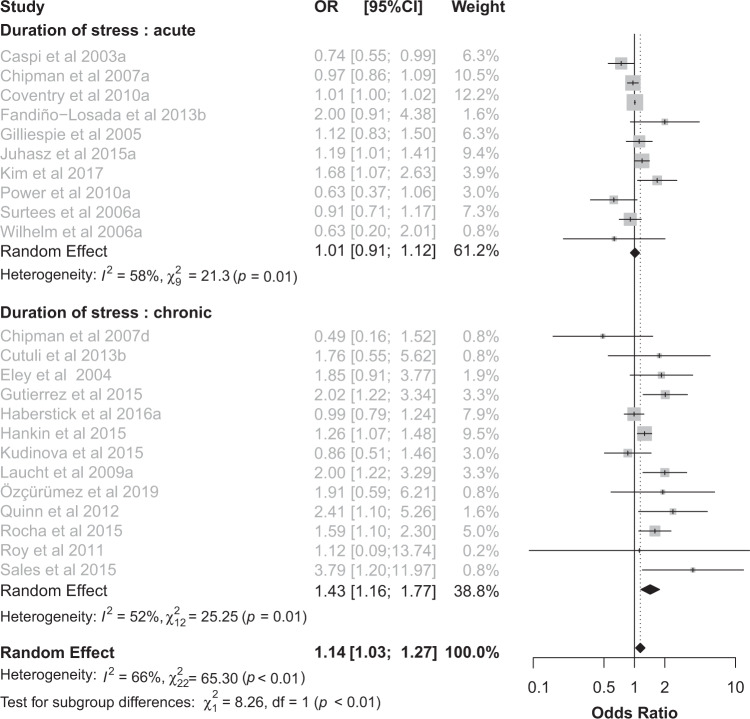
The duration of stress affects the 5-HTTLPR x stress interaction. Forest plot (OR and 95% CI) for 22 studies assessing the relationship between 5-HTTLPR, stress and depression stratified by duration of stress, chronic vs. acute. The area of each square is proportional to the study weight in the analysis. The diamond represents pooled estimates from random-effects meta-analysis. Dashed line represents the overall effect. OR Odds Ratio, CI confidence interval.

Overall, a significant 5-HTTLPR x stress interaction on depression was found (OR 1.14, 95% CI 1.03–1.27, *p* = 0.01; *n* = 22 studies yielding 23 effect sizes, totaling 27,383 participants). There was moderate heterogeneity between studies (*I*^2^ = 66%,  χ2 = 65.30, *p* < 0.0001, Fig. 2).

### Duration of stress (chronic vs. acute)

When stratifying studies for duration of stress (i.e., chronic vs. acute), the effect of 5-HTTLPR on depressive outcome was statistically significant only in interaction with chronic, but not acute, stress (respectively, OR 1.43, 95% CI 1.16–1.77, *p* < 0.001, *I*^2^ = 52%,  χ2 = 25.25, *p* = 0.01 and OR 1.01, 95% CI 0.91–1.12, *p* = 0.81, *I*^2^ = 58%,  χ2 = 21.3, *p* = 0.01; Fig. 2). The interaction test confirmed a subgroup difference (*p* = 0.004), meaning that the duration of stress significantly affects the effect of the 5-HTTLPR x stress interaction. The sensitivity analysis confirmed a significant 5-HTTLPR x stress effect on depressive outcome in the subgroup of studies on chronic stress only (OR 1.17, 95% CI 1.01–1.34, *p* = 0.03, *I*^2^ = 60%,  χ2 = 35.09, *p* = 0.001), despite the subgroup difference failed to reach statistical significance (*p* = 0.13; Supplementary Table 3).

### Time interval (longer than 1 year vs. shorter than or equal to 1 year)

When stratifying the studies in two subgroups based on the time interval between the end of stress and assessment of depression, we found a significant effect of the 5-HTTLPR x stress interaction on depression risk only for those studies in which the time interval was shorter than or equal to 1 year (OR 1.23, 95% CI 1.03–1.46, *p* = 0.02, *I*^2^ = 67%,  χ2 = 39.35, *p* < 0.01), but not for those with time intervals longer than 1 year (OR 1.07, 95% CI 0.90–1.26, *p* = 0.45, *I*^2^ = 56%,  χ2 = 18.23, *p* = 0.02; Supplementary Fig. 1). The subgroup difference failed to reach statistical significance (*p* = 0.25). The sensitivity analysis considering the longer time intervals revealed a statistically significant subgroup difference (*p* = 0.04) (Supplementary Table 4).

### Chronic stress and time interval

**Fig. 3:**
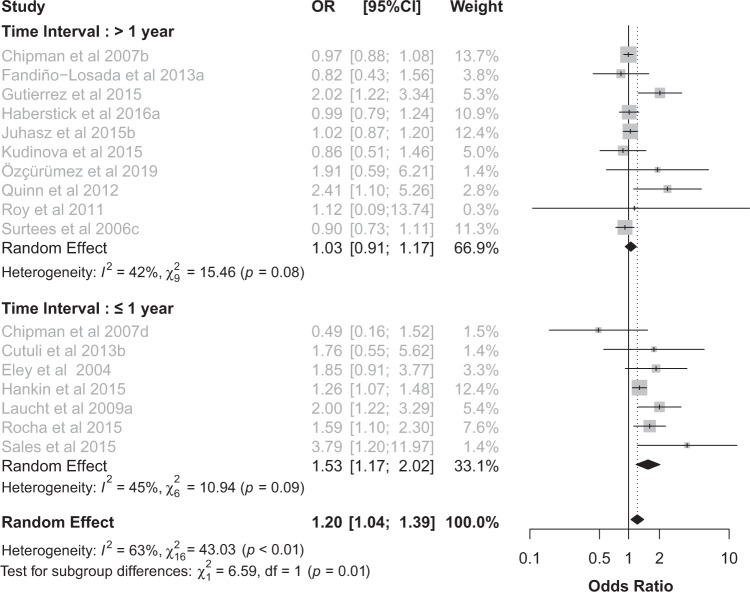
Time interval between the end of the stress and the assessment of depression affects the 5-HTTLPR x stress interaction in chronic stress studies. Forest plot (OR and 95% CI) for 16 studies assessing the relationship between 5-HTTLPR, chronic stress and depression stratified for time intervals (i.e. longer than 1 year, shorter than or equal to 1 year). The area of each square is proportional to the study weight in the analysis. The diamond represents pooled estimates from random-effects meta-analysis. Dashed line represents the overall effect. OR Odds Ratio, CI confidence interval.

To investigate both temporal factors, we stratified only studies investigating chronic stress for time interval (i.e., shorter than or equal to 1 year vs. longer than 1 year), because the analysis of acute stress revealed no effect. The subgroup difference was statistically significant (*p* = 0.01) and a robust 5-HTTLPR x stress interaction was found within 1 year (OR 1.53, 95% CI 1.17–2.02, *p* = 0.002, *I*^2^ = 45%,  χ2 = 10.94, *p* = 0.09) but not for longer time intervals (OR 1.03, 95% CI 0.91–1.17, *p* = 0.64, *I*^2^ = 42%,  χ2 = 15.46, *p* = 0.08; Fig. 3). When the same study reported chronic stress at different time intervals, the shortest interval was included in the analysis. The sensitivity analysis with these alternative choices (Supplementary Table 5) confirmed both the subgroup difference (*p* = 0.02) and the effect of the 5-HTTLPR x stress interaction on depressive outcome only within 1 year (OR 1.53, 95% CI 1.14–2.07, *p* = 0.005, *I*^2^ = 54%,  χ2 = 10.77, *p* = 0.06).

To investigate at higher resolution the length of the time interval in which the 5-HTTLPR x stress interaction was significant, we stratified the studies that considered intervals within 1 year into two time intervals: between 1 year and 6 months and less than 6 months (Supplementary Fig. 2). We found that the interaction was significant in both between 1 year and 6 months (OR 1.30, 95% CI 1.06–1.59 *p* = 0.01, *I*^2^ = 6%, *Q* = 1.07, *p* = 0.30) and within 6 months (OR 1.66, 95% CI 1.08–2.55, *p* = 0.02, *I*^2^ = 43%, χ2 = 7.04, *p* = 0.13). The difference between the subgroups was not significant (*p* = 0.31).

### Tool used for the assessment of depression (clinician-observer scales vs self-reported scales)

When studies were stratified for type of tool used for the assessment of depression—categorized as clinician-observer scales or self-reported scales (Supplementary Table 1), the subgroup difference failed to reach statistical significance (*p* = 0.37; *clinician-observer scales:* OR 1.12, 95% CI 1.00–1.26, *p* = 0.05, *I*^2^ = 69%, χ2 = 45.05, *p* < 0.01; *self-reported scales:* OR 1.32, 95% CI 0.95–1.83, *p* = 0.10, *I*^2^ = 64%, χ2 = 19.26, *p* < 0.01*;* Supplementary Fig. 3). The sensitivity analysis confirmed these results, and the subgroup difference did not reach statistical significance (*p* = 0.53; Supplementary Table 6).

### Quality assessment (risk of bias) and publication bias

Eight of the 22 included studies (describing 23 populations) show moderate risk of bias, 15 studies show low risk, while no study showed high risk (Supplementary Table 7). Visual inspection of funnel plots and the Egger test indicate publication bias (*p* = 0.032; Supplementary Fig. 4).

## Discussion

The present results show an overall evidence for an effect of the 5-HTTLPR x stress interaction on the depression risk in line with some previous studies and meta-analyses [15, 16, 19], but in contrast with others [17, 18]. Such discrepancy, on the one hand, might be due to the different cohorts of patients investigated or to the different studies included in the meta-analyses. On the other, it corroborates the view that a moderating factor determines the outcome of the 5-HTTLPR x stress interaction. We identified time as a key moderating factor and showed that temporal factors contribute to the outcome of the interaction, reconciling the potential discrepant results reported in the literature.

The first temporal factor affecting the 5-HTTLPR x stress interaction was the duration of stress. After stratifying for chronic and acute stress, a significant subgroup difference was found and a robust effect of the interaction between the polymorphism and chronic, but not acute, stress on depression risk emerged (Fig. 2). This is in line with previous analyses that assessed the relevance of different types of stress and found only marginal evidence for a significant association between 5-HTTLPR with acute stressful events [16]. This factor may also account for the discrepancy among previous meta-analyses since some included almost exclusively studies on acute stress [17, 18]. The relevance of chronicity is also in line with the study by Caspi and collaborators who reported a clear dose-dependent effect of the number of stressful life events suffered by patients in making the effect of the 5-HTTLPR emerge [7]. Accordingly, the distinction between chronic and acute stresses is widely acknowledged in the psychiatric field and a larger impact of chronic stress on the risk of depression has been reported [1, 57].

A further temporal factor moderating the 5-HTTLPR x stress interaction was the length of the time interval between the end of stress and assessment of depression. When stratifying for time interval, a robust interaction between the polymorphism and stress emerged only at intervals shorter than or equal to 1 year (Supplementary Fig. 1). The relevance of time in moderating the 5-HTTLPR x stress interaction emerged even more clearly when considering both temporal factors: duration of stress and time interval. When including only chronic stress studies and stratifying for time interval, a significant subgroup difference was found, and the effect of the interaction emerged only within time intervals shorter than 1 year (Fig. 3).The highly significant heterogeneity in the overall group of studies on chronic stress was no more significant after subgrouping for the two time intervals, corroborating the hypothesis that the 5-HTTLPR x stress interaction produces different and well defined effects on the risk of depression before and after 1 year from the end of the stress.

The duration of the time interval in which the effect of the interaction is strongest has been previously debated. Some authors proposed that such interval lasts from one to 3 months [30] while others found an interval up to 6 months [31, 32] or up to 1 year [33]. To better define such duration, we analyzed the 5-HTTLPR x stress interaction stratifying the studies that considered intervals within 1 year into two intervals: shorter than 6 months and between 6 months and 1 year. We found no subgroup difference and the interaction was significant in both intervals, confirming that the effects are detectable up to 1 year from the end of stress. However, the heterogeneity in both two subgroups was reduced and the OR point estimate appears inversely proportional to the length of the time interval, supporting the view that the interaction may produce different effects at subsequent time intervals within 1 year. Since only two studies report data related to the time interval between 6 months and 1 year, this warrants further analyses.

The distinction between clinician-observer scales and self-reported scales has been widely shown to be relevant in assessing psychiatric disorders [58, 59]. Therefore, we stratified the studies for these classes of tools categorized as clinician-observer scales vs. self-reported scales (Supplementary Table 1). In both groups of studies, no significant 5-HTTLPR x stress interaction on depression risk was found; however, the group of studies exploiting clinician-observer scales was close to the statistical significance, suggesting that this class of tools could be more reliable in revealing gene x environment interaction effects (Supplementary Fig. 3).

Dynamic processes are highly relevant in the neuroscientific and psychiatric fields. Biological characteristics, from psychological traits to neural properties, are increasingly acknowledged as factors changing over time and being dependent on the context [60–62]. Growing evidence points to neural plasticity—the capability of the brain to change its function and structure—as being a dynamic process producing effects that differ in time and according to the environment [22]. Indeed, an enhancement of neural plasticity does not produce a univocal outcome but opens a window of opportunity for a change at the neural and behavioral levels [63]. Accordingly, plasticity has been proposed to act through permissive causality since it affects the duration and likelihood of a change to occur but does not define the form that such change should take [22]. The 5-HTTLPR regulates neural and behavioral plasticity as shown both at preclinical [64, 65] and clinical [23, 66–68] levels. The molecular mechanisms linking 5-HTTLPR and serotonin to plasticity regulation have been proposed to include the modulation of neuronal firing properties, affecting signal processing and long-term synaptic plasticity, and the neurogenesis [69, 70]. The important involvement of the 5-HTTLPR in plasticity suggests that the results obtained in this study can be interpreted from the perspective of a dynamic process. This implies that the different levels of plasticity associated to the two alleles of the 5-HTTLPR translate into different rates of change in brain function and behavioral outcome in response to stress. Accordingly, the 5-HTTLPR x stress interaction emerges almost exclusively following chronic stress because the different outcomes associated to the different plasticity levels characterizing the s- and l- carriers become magnified as a function of the duration of the stress. By contrast, with acute stressful events there is no time for this difference to emerge. In addition, the loss of the effect of the interaction after time intervals longer than 1 year from the end of stress can be explained by the higher plasticity level shown by s-carriers, compared to l-carriers, that make them at higher risk but also more able to recover from depression [23, 71]. Therefore, at short time intervals from the end of the stress, s-carriers show an increased risk of depression. However, since they are also more able to recover, at long-time intervals the difference between s- and l-carriers disappears and the 5-HTTLPR x stress interaction is no more significant.

The view of neural plasticity as a dynamic process has been already successfully exploited to explain the action of selective serotonin reuptake inhibitors (SSRIs) [63, 72, 73], which have a molecular mechanism overlapping with that of 5-HTTLPR [6]. The undirected susceptibility to change model posits that SSRI treatment does not affect mood per se but, by increasing neural plasticity, increases the likelihood of a change in mood that is driven by the quality of the living conditions and defined by time [63]. Recent studies have demonstrated such a hypothesis both at preclinical [72, 74, 75] and clinical [67, 68, 76, 77] levels. The dynamic nature of SSRI outcome has been described also for endpoints different from depression, such as vulnerability to obesity [78], and growing evidence suggests the same conceptual approach applies to interpret the action of other classes of drugs regulating plasticity, such as psychedelics [79, 80]. The present results indicate that the same theoretical model can be applied to explain also 5-HTTLPR outcome and, in a broader perspective, the effects of interventions affecting brain plasticity. In addition, it can be also relevant to assess the time-dependent effects of responses such as avoidance behavior or anxiety, as this polymorphism was originally reported to be associated with anxiety-related traits in both human subjects and animal models [5, 81]. To this aim, further studies assessing the anxiety response according to the 5-HTTLPR at different time intervals are warranted.

Different limitations of this study should be acknowledged. We excluded all studies that used statistical approaches different from odds ratio or logistic regression, reducing the number of available studies. Although we stratified risk estimates for depression diagnosis at different times from the end of stress, we could not reliably investigate all possible intervals because of the irregular distribution of the time intervals investigated in the published studies. In addition, most of the studies included in the present analyses considered as chronic stress only early life adversity, making further investigations of the 5-HTTLPR x chronic stress interaction in adulthood warranted. However, several studies that have not been included in the present analysis because of differences in the statistical approaches suggest that the results here reported can be generalized to other age stages [10, 36, 66, 82]. Though for this study we considered chronic and acute stress to differ only for the duration, there might be differences in the quality and quantity of the stressor itself. Thus, further studies are warranted to better detail the differences between acute and chronic stress. Finally, though there is no univocal definition of childhood duration, in the present study we set its age limit at 18 years as indicated by the World Health Organization [83]. This can represent a limitation when young adults live in their family environment and are still subjected to abuse/maltreatment after the 18 years of age.

In conclusion, our findings support the 5-HTTLPR x stress interaction hypothesis and show that the effect of the interaction on depression risk is significantly moderated by time. This is corroborated by the fact that two independent temporal factors, duration of stress and time interval between end of stress and assessment of depression, affect the outcome of the interaction in a coherent and complementary fashion. In addition, given the key role played by the 5-HTTLPR in regulating neural plasticity, these findings fit and corroborate the view of neural plasticity as a dynamic process. A critical implication is that the two alleles of the 5-HTTLPR, which are associated to different plasticity levels, do not univocally lead to a beneficial or detrimental outcome per se, but their value must be estimated according to temporal factors and the context [62]. This view potentially reconciles the discrepancies on the effect of the 5-HTTLPR x stress interaction reported in the literature. In addition, it explains apparently discordant findings concerning vulnerability and recovery associated with 5-HTTLPR alleles: s-carriers are both at higher risk for depression within 1 year and have depressive episodes that are about 20 weeks shorter compared to l-carriers [71, 84]. It also justifies the broad effects of this polymorphism that concern not a single psychopathology but many mental disorders including mood disorders, obsessive-compulsive disorder and autism [34, 85–87]. Indeed, the view of plasticity as a dynamic process involved in the shift between a pathological and a healthy state makes it a key process in almost all psychiatric disorders. This view might also inform other studies on the outcome of gene x environment interactions involving other gene polymorphisms know to regulate neural plasticity, such as the dopamine receptor D4 and the brain-derived neurotrophic factor genes [23, 88, 89]. Finally, it is worth noting that the role of 5-HTTLPR in plasticity suggests novel approaches, not only to predict risk of depression, but also to develop personalized preventive and therapeutic interventions whose effectiveness differs between s- and l-carriers depending on time elapsed from stress [90–92]. Overall, our findings point out the need of a gene x environment x time interaction to understand how brain activity evolves over time to promote mental health.

## Supplementary information

Supplementary Material

## Data Availability

All R codes used to generate the meta-analysis results can be obtained from the authors upon request.

## References

[1] Hammen C, Kim EY, Eberhart NK, Brennan PA (2009). Chronic and acute stress and the prediction of major depression in women. Depression Anxiety.

[2] Kendler KS, Kessler RC, Walters EE, MacLean C, Neale MC, Heath AC (1995). Stressful life events, genetic liability, and onset of an episode of major depression in women. Am J Psychiatry.

[3] Southwick SM, Charney DS (2012). The science of resilience: implications for the prevention and treatment of depression. Science.

[4] Caspi A, Moffitt TE (2006). Gene-environment interactions in psychiatry: joining forces with neuroscience. Nat Rev Neurosci.

[5] Lesch KP, Bengel D, Heils A, Sabol SZ, Greenberg BD, Petri S (1996). Association of anxiety-related traits with a polymorphism in the serotonin transporter gene regulatory region. Science.

[6] Murphy DL, Lesch KP (2008). Targeting the murine serotonin transporter: insights into human neurobiology. Nat Rev Neurosci.

[7] Caspi A, Sugden K, Moffitt TE, Taylor A, Craig IW, Harrington H (2003). Influence of life stress on depression: moderation by a polymorphism in the 5-HTT gene. Science.

[8] Brown GW, Ban M, Craig TK, Harris TO, Herbert J, Uher R (2013). Serotonin transporter length polymorphism, childhood maltreatment, and chronic depression: a specific gene-environment interaction. Depression Anxiety.

[9] Ming QS, Zhang Y, Chai QL, Chen HY, Hou CJ, Wang MC (2013). Interaction between a serotonin transporter gene promoter region polymorphism and stress predicts depressive symptoms in Chinese adolescents: a multi-wave longitudinal study. BMC Psychiatry.

[10] Otte C, McCaffery J, Ali S, Whooley MA (2007). Association of a serotonin transporter polymorphism (5-HTTLPR) with depression, perceived stress, and norepinephrine in patients with coronary disease: the Heart and Soul Study. Am J Psychiatry.

[11] Stefanis NC, Mandelli L, Hatzimanolis A, Zaninotto L, Smyrnis N, Avramopoulos D (2011). Serotonin transporter gene variants and prediction of stress-induced risk for psychological distress. Genes Brain Behav.

[12] Chorbov VM, Lobos EA, Todorov AA, Heath AC, Botteron KN, Todd RD (2007). Relationship of 5-HTTLPR genotypes and depression risk in the presence of trauma in a female twin sample. Am J Med Genet B Neuropsychiatr Genet.

[13] Middeldorp CM, de Geus EJ, Beem AL, Lakenberg N, Hottenga JJ, Slagboom PE (2007). Family based association analyses between the serotonin transporter gene polymorphism (5-HTTLPR) and neuroticism, anxiety and depression. Behav Genet.

[14] Surtees PG, Wainwright NW, Willis-Owen SA, Luben R, Day NE, Flint J (2006). Social adversity, the serotonin transporter (5-HTTLPR) polymorphism and major depressive disorder. Biol Psychiatry.

[15] Bleys D, Luyten P, Soenens B, Claes S (2018). Gene-environment interactions between stress and 5-HTTLPR in depression: a meta-analytic update. J Affect Disord.

[16] Karg K, Burmeister M, Shedden K, Sen S (2011). The serotonin transporter promoter variant (5-HTTLPR), stress, and depression meta-analysis revisited: evidence of genetic moderation. Arch Gen Psychiatry.

[17] Munafo MR, Durrant C, Lewis G, Flint J (2009). Gene X environment interactions at the serotonin transporter locus. Biol Psychiatry.

[18] Risch N, Herrell R, Lehner T, Liang KY, Eaves L, Hoh J (2009). Interaction between the serotonin transporter gene (5-HTTLPR), stressful life events, and risk of depression: a meta-analysis. JAMA.

[19] Sharpley CF, Palanisamy SK, Glyde NS, Dillingham PW, Agnew LL (2014). An update on the interaction between the serotonin transporter promoter variant (5-HTTLPR), stress and depression, plus an exploration of non-confirming findings. Behavioural Brain Res.

[20] Culverhouse RC, Saccone NL, Horton AC, Ma Y, Anstey KJ, Banaschewski T (2018). Collaborative meta-analysis finds no evidence of a strong interaction between stress and 5-HTTLPR genotype contributing to the development of depression. Mol Psychiatry.

[21] Ellis BJ, Boyce WT (2011). Differential susceptibility to the environment: toward an understanding of sensitivity to developmental experiences and context. Dev Psychopathol.

[22] Branchi I, Giuliani A (2021). Shaping therapeutic trajectories in mental health: Instructive vs. permissive causality. Eur Neuropsychopharmacol..

[23] Belsky J, Jonassaint C, Pluess M, Stanton M, Brummett B, Williams R (2009). Vulnerability genes or plasticity genes?. Mol Psychiatry.

[24] Ouzzani M, Hammady H, Fedorowicz Z, Elmagarmid A (2016). Rayyan-a web and mobile app for systematic reviews. Syst Rev.

[25] The Joanna Briggs Institute TJB. *Joanna Briggs Institute Reviewers’ Manual: 2014 edition*. 2014 edn. The Joanna Briggs Institute: Adelaide, Australia, 2014.

[26] Higgins JPT, Thomas J, Chandler J, Cumpston M, Li T, Page MJ, et al. *Cochrane Handbook for Systematic Reviews of Interventions*. 2nd Edition edn. John Wiley & Sons: Chichester (UK), 2019.

[27] Goplen CM, Verbeek W, Kang SH, Jones CA, Voaklander DC, Churchill TA (2019). Preoperative opioid use is associated with worse patient outcomes after Total joint arthroplasty: a systematic review and meta-analysis. BMC Musculoskelet Disord.

[28] Harrer M, Cuijpers P, Furukawa TA, Ebert DD (2021). Doing meta-analysis with R: a hands-on guide.

[29] Higgins JP, Thompson SG, Deeks JJ, Altman DG (2003). Measuring inconsistency in meta-analyses. Bmj.

[30] Kendler KS, Karkowski LM, Prescott CA (1998). Stressful life events and major depression: risk period, long-term contextual threat, and diagnostic specificity. J Nerv Ment Dis.

[31] Boyce P, Parker G, Barnett B, Cooney M, Smith F (1991). Personality as a vulnerability factor to depression. Br J Psychiatry.

[32] Brugha TS, Bebbington PE, Sturt E, MacCarthy B, Wykes T (1990). The relation between life events and social support networks in a clinically depressed cohort. Soc psychiatry Psychiatr Epidemiol.

[33] Solomon Z, Bromet E (1982). The role of social factors in affective disorder: an assessment of the vulnerability model of Brown and this colleagues. Psychol Med.

[34] Wang R, Ware JH (2013). Detecting moderator effects using subgroup analyses. Prev Sci.

[35] Ancelin ML, Scali J, Norton J, Ritchie K, Dupuy AM, Chaudieu I (2017). Heterogeneity in HPA axis dysregulation and serotonergic vulnerability to depression. Psychoneuroendocrinology.

[36] Artero S, Touchon J, Dupuy AM, Malafosse A, Ritchie K (2011). War exposure, 5-HTTLPR genotype and lifetime risk of depression. Br J Psychiatry.

[37] Power T, Stewart R, Ancelin ML, Jaussent I, Malafosse A, Ritchie K (2010). 5-HTTLPR genotype, stressful life events and late-life depression: no evidence of interaction in a French population. Neurobiol Aging.

[38] Chipman P, Jorm AF, Prior M, Sanson A, Smart D, Tan X (2007). No interaction between the serotonin transporter polymorphism (5-HTTLPR) and childhood adversity or recent stressful life events on symptoms of depression: results from two community surveys. Am J Med Genet B Neuropsychiatr Genet.

[39] Coventry WL, James MR, Eaves LJ, Gordon SD, Gillespie NA, Ryan L (2010). Do 5HTTLPR and stress interact in risk for depression and suicidality? Item response analyses of a large sample. Am J Med Genet B Neuropsychiatr Genet.

[40] Cutuli JJ, Raby KL, Cicchetti D, Englund MM, Egeland B (2013). Contributions of maltreatment and serotonin transporter genotype to depression in childhood, adolescence, and early adulthood. J Affect Disord.

[41] Eley TC, Sugden K, Corsico A, Gregory AM, Sham P, McGuffin P (2004). Gene-environment interaction analysis of serotonin system markers with adolescent depression. Mol Psychiatry.

[42] Fandiño-Losada A, Wei Y, Aberg E, Sjoholm LK, Lavebratt C, Forsell Y (2013). Influence of serotonin transporter promoter variation on the effects of separation from parent/partner on depression. J Affect Disord.

[43] Gillespie NA, Whitfield JB, Williams B, Heath AC, Martin NG (2005). The relationship between stressful life events, the serotonin transporter (5-HTTLPR) genotype and major depression. Psychol Med.

[44] Gutierrez B, Bellon JA, Rivera M, Molina E, King M, Marston L (2015). The risk for major depression conferred by childhood maltreatment is multiplied by BDNF and SERT genetic vulnerability: a replication study. J Psychiatry Neurosci.

[45] Haberstick BC, Boardman JD, Wagner B, Smolen A, Hewitt JK, Killeya-Jones LA (2016). Depression, stressful life events, and the impact of variation in the serotonin transporter: findings from the national longitudinal study of adolescent to adult health (add health). PloS One.

[46] Hankin BL, Young JF, Abela JR, Smolen A, Jenness JL, Gulley LD (2015). Depression from childhood into late adolescence: Influence of gender, development, genetic susceptibility, and peer stress. J Abnorm Psychol.

[47] Juhasz G, Gonda X, Hullam G, Eszlari N, Kovacs D, Lazary J (2015). Variability in the effect of 5-HTTLPR on depression in a large European population: the role of age, symptom profile, type and intensity of life stressors. PloS One.

[48] Kim JM, Stewart R, Kang HJ, Bae KY, Kim SW, Shin IS (2017). Depression following acute coronary syndrome: time-specific interactions between stressful life events, social support deficits, and 5-HTTLPR. Psychother Psychosom.

[49] Kudinova AY, Gibb BE, McGeary JE, Knopik VS (2015). Brain derived neurotrophic factor (BDNF) polymorphism moderates the interactive effect of 5-HTTLPR polymorphism and childhood abuse on diagnoses of major depression in women. Psychiatry Res.

[50] Laucht M, Treutlein J, Blomeyer D, Buchmann AF, Schmid B, Becker K (2009). Interaction between the 5-HTTLPR serotonin transporter polymorphism and environmental adversity for mood and anxiety psychopathology: evidence from a high-risk community sample of young adults. Int J Neuropsychopharmacol.

[51] Özçürümez G, Yurdakul HT, Terzi Y, Direk N, Essizoglu A, Sahin F (2019). No interaction between childhood maltreatment and serotonin transporter gene in recurrent major depressive disorder: a clinical sample. Noro Psikiyatr Ars.

[52] Quinn CR, Dobson-Stone C, Outhred T, Harris A, Kemp AH (2012). The contribution of BDNF and 5-HTT polymorphisms and early life stress to the heterogeneity of major depressive disorder: a preliminary study. Aust N. Z J Psychiatry.

[53] Rocha TB, Hutz MH, Salatino-Oliveira A, Genro JP, Polanczyk GV, Sato JR (2015). Gene-environment interaction in youth depression: replication of the 5-HTTLPR moderation in a diverse setting. Am J Psychiatry.

[54] Roy A, Roy M, Goldman D (2011). Childhood trauma and depressive symptoms in type 1 diabetes. J Clin psychiatry.

[55] Sales JM, Brown JL, Swartzendruber AL, Smearman EL, Brody GH, DiClemente R (2015). Genetic sensitivity to emotional cues, racial discrimination and depressive symptoms among African–American adolescent females. Front Psychol.

[56] Wilhelm K, Mitchell PB, Niven H, Finch A, Wedgwood L, Scimone A (2006). Life events, first depression onset and the serotonin transporter gene. Br J Psychiatry.

[57] McGonagle KA, Kessler RC (1990). Chronic stress, acute stress, and depressive symptoms. Am J Community Psychol.

[58] Möller HJ (2000). Rating depressed patients: observer- vs self-assessment. Eur Psychiatry: J Assoc Eur Psychiatrists.

[59] Uher R, Perlis RH, Placentino A, Dernovsek MZ, Henigsberg N, Mors O (2012). Self-report and clinician-rated measures of depression severity: can one replace the other?. Depression Anxiety.

[60] Goel A, Buonomano DV (2014). Timing as an intrinsic property of neural networks: evidence from in vivo and in vitro experiments. Philos Trans R Soc Lond B Biol Sci.

[61] Kuper N, Modersitzki N, Phan LV, Rauthmann JF (2021). The dynamics, processes, mechanisms, and functioning of personality: an overview of the field. Br J Psychol.

[62] Branchi I (2022). Recentering neuroscience on behavior: the interface between brain and environment is a privileged level of control of neural activity. Neurosci Biobehav Rev.

[63] Branchi I (2011). The double edged sword of neural plasticity: increasing serotonin levels leads to both greater vulnerability to depression and improved capacity to recover. Psychoneuroendocrinology.

[64] Karabeg MM, Grauthoff S, Kollert SY, Weidner M, Heiming RS, Jansen F (2013). 5-HTT deficiency affects neuroplasticity and increases stress sensitivity resulting in altered spatial learning performance in the Morris water maze but not in the Barnes maze. PloS One.

[65] Nietzer SL, Bonn M, Jansen F, Heiming RS, Lewejohann L, Sachser N (2011). Serotonin transporter knockout and repeated social defeat stress: impact on neuronal morphology and plasticity in limbic brain areas. Behavioural Brain Res.

[66] Haase CM, Saslow LR, Bloch L, Saturn SR, Casey JJ, Seider BH (2013). The 5-HTTLPR polymorphism in the serotonin transporter gene moderates the association between emotional behavior and changes in marital satisfaction over time. Emotion.

[67] Klobl M, Seiger R, Vanicek T, Handschuh P, Reed MB, Spurny-Dworak B (2021). Escitalopram modulates learning content-specific neuroplasticity of functional brain networks. Neuroimage.

[68] Reed MB, Klobl M, Godbersen GM, Handschuh PA, Ritter V, Spurny-Dworak B (2022). Serotonergic modulation of effective connectivity in an associative relearning network during task and rest. Neuroimage.

[69] Normann C, Clark K (2005). Selective modulation of Ca(2+) influx pathways by 5-HT regulates synaptic long-term plasticity in the hippocampus. Brain Res.

[70] Schmitt A, Benninghoff J, Moessner R, Rizzi M, Paizanis E, Doenitz C (2007). Adult neurogenesis in serotonin transporter deficient mice. J Neural Transm.

[71] Eaton WW, Shao H, Nestadt G, Lee HB, Bienvenu OJ, Zandi P (2008). Population-based study of first onset and chronicity in major depressive disorder. Arch Gen Psychiatry.

[72] Alboni S, Van Dijk RM, Poggini S, Milior G, Perrotta M, Drenth T (2017). Fluoxetine effects on molecular, cellular and behavioral endophenotypes of depression are driven by the living environment. Mol Psychiatry.

[73] Branchi I (2022). Plasticity in mental health: A network theory. Neurosci Biobehav Rev.

[74] Branchi I, Santarelli S, Capoccia S, Poggini S, D’Andrea I, Cirulli F (2013). Antidepressant treatment outcome depends on the quality of the living environment: a pre-clinical investigation in mice. PloS One.

[75] Poggini S, Matte Bon G, Golia MT, Ciano Albanese N, Viglione A, Poleggi A (2021). Selecting antidepressants according to a drug-by-environment interaction: a comparison of fluoxetine and minocycline effects in mice living either in enriched or stressful conditions. Behavioural Brain Res.

[76] Chiarotti F, Viglione A, Giuliani A, Branchi I (2017). Citalopram amplifies the influence of living conditions on mood in depressed patients enrolled in the STAR*D study. Transl Psychiatry.

[77] Viglione A, Chiarotti F, Poggini S, Giuliani A, Branchi I (2019). Predicting antidepressant treatment outcome based on socioeconomic status and citalopram dose. Pharmacogenomics J.

[78] Mastronardi C, Paz-Filho GJ, Valdez E, Maestre-Mesa J, Licinio J, Wong ML (2011). Long-term body weight outcomes of antidepressant-environment interactions. Mol Psychiatry.

[79] Carhart-Harris RL, Roseman L, Haijen E, Erritzoe D, Watts R, Branchi I (2018). Psychedelics and the essential importance of context. J Psychopharmacol.

[80] Lepow L, Morishita H, Yehuda R (2021). Critical Period Plasticity as a Framework for Psychedelic-Assisted Psychotherapy. Front Neurosci.

[81] Lesch KP, Meyer J, Glatz K, Flugge G, Hinney A, Hebebrand J (1997). The 5-HT transporter gene-linked polymorphic region (5-HTTLPR) in evolutionary perspective: alternative biallelic variation in rhesus monkeys. Rapid communication. J Neural Transm.

[82] Kilpatrick DG, Koenen KC, Ruggiero KJ, Acierno R, Galea S, Resnick HS (2007). The serotonin transporter genotype and social support and moderation of posttraumatic stress disorder and depression in hurricane-exposed adults. Am J Psychiatry.

[83] WHO. Child maltreatment. World Health Organization. 2020; https://www.who.int/news-room/fact-sheets/detail/child-maltreatment.

[84] Myung W, Lim SW, Kim J, Lee Y, Song J, Chang KW (2010). Serotonin transporter gene polymorphisms and chronic illness of depression. J Korean Med Sci.

[85] Cho HJ, Meira-Lima I, Cordeiro Q, Michelon L, Sham P, Vallada H (2005). Population-based and family-based studies on the serotonin transporter gene polymorphisms and bipolar disorder: a systematic review and meta-analysis. Mol Psychiatry.

[86] Nunez-Rios DL, Chaskel R, Lopez A, Galeano L, Lattig MC (2020). The role of 5-HTTLPR in autism spectrum disorder: new evidence and a meta-analysis of this polymorphism in Latin American population with psychiatric disorders. PloS One.

[87] Rotondo A, Mazzanti C, Dell’Osso L, Rucci P, Sullivan P, Bouanani S (2002). Catechol o-methyltransferase, serotonin transporter, and tryptophan hydroxylase gene polymorphisms in bipolar disorder patients with and without comorbid panic disorder. Am J Psychiatry.

[88] Pearson-Fuhrhop KM, Kleim JA, Cramer SC (2009). Brain plasticity and genetic factors. Top Stroke Rehabil.

[89] Kleim JA, Chan S, Pringle E, Schallert K, Procaccio V, Jimenez R (2006). BDNF val66met polymorphism is associated with modified experience-dependent plasticity in human motor cortex. Nat Neurosci.

[90] Smeraldi E, Benedetti F, Zanardi R (2002). Serotonin transporter promoter genotype and illness recurrence in mood disorders. Eur Neuropsychopharmacol.

[91] Rausch JL, Johnson ME, Fei YJ, Li JQ, Shendarkar N, Hobby HM (2002). Initial conditions of serotonin transporter kinetics and genotype: influence on SSRI treatment trial outcome. Biol Psychiatry.

[92] Vai B, Serretti A, Poletti S, Mascia M, Lorenzi C, Colombo C (2020). Cortico-limbic functional connectivity mediates the effect of early life stress on suicidality in bipolar depressed 5-HTTLPR*s carriers. J Affect Disord.

