# Supplementary material for: Time moderates the interplay between 5-HTTLPR and stress on depression risk: gene x environment interaction as a dynamic process
Source: Transl Psychiatry. 2022 Jul 11;12:274. doi: 10.1038/s41398-022-02035-4 (PMC9276704; doi:10.1038/s41398-022-02035-4)

Supplementary

Supplementary Methods 1. PRISMA guidelines for meta-analysis and systematic reviews 2

Supplementary Methods 2. Full search terms 5

Supplementary Table 1. Classification of diagnostic tools 7

Supplementary Methods 3. Formulas 9

Supplementary Table 2. Studies excluded in meta-analysis reporting overlapped population 10

Supplementary Table 3. Sensitivity analysis for stress 11

Supplementary Figure 1. Time interval between the end of the stress and the assessment of depression 12

Supplementary Table 4. Sensitivity analysis for time interval 13

Supplementary Table 5. Sensitivity analysis for time interval (only chronic stress) 14

Supplementary Figure 2. Time interval between the end of the stress and the assessment of depression only in chronic stress studies 15

Supplementary Figure 3. Diagnostic tool 16

Supplementary Table 6. Sensitivity analysis for diagnostic tool 17

Supplementary Table 7. JBI critical appraisal tool 18

Supplementary Figure 4. Funnel Plot 19

Supplementary Methods 1. PRISMA guidelines for meta-analysis and systematic reviews

Section and Topic	Item #	Checklist item	Location where item is reported
**TITLE**			
Title	1	Identify the report as a systematic review.	1
**ABSTRACT**			
Abstract	2	See the PRISMA 2020 for Abstracts checklist.	2
**INTRODUCTION**			
Rationale	3	Describe the rationale for the review in the context of existing knowledge.	3-4
Objectives	4	Provide an explicit statement of the objective(s) or question(s) the review addresses.	3-4
**METHODS**			
Eligibility criteria	5	Specify the inclusion and exclusion criteria for the review and how studies were grouped for the syntheses.	5
Information sources	6	Specify all databases, registers, websites, organisations, reference lists and other sources searched or consulted to identify studies. Specify the date when each source was last searched or consulted.	5
Search strategy	7	Present the full search strategies for all databases, registers and websites, including any filters and limits used.	5
Selection process	8	Specify the methods used to decide whether a study met the inclusion criteria of the review, including how many reviewers screened each record and each report retrieved, whether they worked independently, and if applicable, details of automation tools used in the process.	5
Data collection process	9	Specify the methods used to collect data from reports, including how many reviewers collected data from each report, whether they worked independently, any processes for obtaining or confirming data from study investigators, and if applicable, details of automation tools used in the process.	5
Data items	10a	List and define all outcomes for which data were sought. Specify whether all results that were compatible with each outcome domain in each study were sought (e.g. for all measures, time points, analyses), and if not, the methods used to decide which results to collect.	5
	10b	List and define all other variables for which data were sought (e.g. participant and intervention characteristics, funding sources). Describe any assumptions made about any missing or unclear information.	6
Study risk of bias assessment	11	Specify the methods used to assess risk of bias in the included studies, including details of the tool(s) used, how many reviewers assessed each study and whether they worked independently, and if applicable, details of automation tools used in the process.	7
Effect measures	12	Specify for each outcome the effect measure(s) (e.g. risk ratio, mean difference) used in the synthesis or presentation of results.	6
	13a	Describe the processes used to decide which studies were eligible for each synthesis (e.g. tabulating the study	5

Synthesis methods		intervention characteristics and comparing against the planned groups for each synthesis (item #5)).	
	13b	Describe any methods required to prepare the data for presentation or synthesis, such as handling of missing summary statistics, or data conversions.	5-8
	13c	Describe any methods used to tabulate or visually display results of individual studies and syntheses.	8
	13d	Describe any methods used to synthesize results and provide a rationale for the choice(s). If meta-analysis was performed, describe the model(s), method(s) to identify the presence and extent of statistical heterogeneity, and software package(s) used.	8
	13e	Describe any methods used to explore possible causes of heterogeneity among study results (e.g. subgroup analysis, meta-regression).	8
	13f	Describe any sensitivity analyses conducted to assess robustness of the synthesized results.	8
Reporting bias assessment	14	Describe any methods used to assess risk of bias due to missing results in a synthesis (arising from reporting biases).	n.a
Certainty assessment	15	Describe any methods used to assess certainty (or confidence) in the body of evidence for an outcome.	8-9
**RESULTS**			
Study selection	16a	Describe the results of the search and selection process, from the number of records identified in the search to the number of studies included in the review, ideally using a flow diagram.	10
	16b	Cite studies that might appear to meet the inclusion criteria, but which were excluded, and explain why they were excluded.	Table 1; Supplementary Table 2
Study characteristics	17	Cite each included study and present its characteristics.	Table 1
Risk of bias in studies	18	Present assessments of risk of bias for each included study.	12
Results of individual studies	19	For all outcomes, present, for each study: (a) summary statistics for each group (where appropriate) and (b) an effect estimate and its precision (e.g. confidence/credible interval), ideally using structured tables or plots.	Figure 2-3
Results of syntheses	20a	For each synthesis, briefly summarise the characteristics and risk of bias among contributing studies.	Figure 2-3; Supplementary: Figure 1-2-3, Table 7
	20b	Present results of all statistical syntheses conducted. If meta-analysis was done, present for each the summary estimate and its precision (e.g. confidence/credible interval) and measures of statistical heterogeneity. If comparing groups, describe the direction of the effect.	10-12
	20c	Present results of all investigations of possible causes of heterogeneity among study results.	Supplementary Table 3-4-5
	20d	Present results of all sensitivity analyses conducted to assess the robustness of the synthesized results.	Supplementary Table 3-4-5

Reporting biases	21	Present assessments of risk of bias due to missing results (arising from reporting biases) for each synthesis assessed.	n.a
Certainty of evidence	22	Present assessments of certainty (or confidence) in the body of evidence for each outcome assessed.	n.a
**DISCUSSION**			
Discussion	23a	Provide a general interpretation of the results in the context of other evidence.	13-15
	23b	Discuss any limitations of the evidence included in the review.	15
	23c	Discuss any limitations of the review processes used.	15
	23d	Discuss implications of the results for practice, policy, and future research.	16-17
**OTHER INFORMATION**			
Registration and protocol	24a	Provide registration information for the review, including register name and registration number, or state that the review was not registered.	5
	24b	Indicate where the review protocol can be accessed, or state that a protocol was not prepared.	
	24c	Describe and explain any amendments to information provided at registration or in the protocol.	
Support	25	Describe sources of financial or non-financial support for the review, and the role of the funders or sponsors in the review.	
Competing interests	26	Declare any competing interests of review authors.	
Availability of data, code and other materials	27	Report which of the following are publicly available and where they can be found: template data collection forms; data extracted from included studies; data used for all analyses; analytic code; any other materials used in the review.	9

From: Page MJ, McKenzie JE, Bossuyt PM, Boutron I, Hoffmann TC, Mulrow CD, et al. The PRISMA 2020 statement: an updated guideline for reporting systematic reviews. BMJ 2021;372:n71. doi: 10.1136/bmj.n71

Supplementary Methods 2. Full search terms

EMBASE

(maltreatment:ti,ab,kw OR trauma*:ti,ab,kw OR stress*:ti,ab,kw OR 'adverse':ti,ab,kw OR 'life event*':ti,ab,kw OR 'genetic moderation':ti,ab,kw OR 'gene-environment':ti,ab,kw OR 'gene-by-environment':ti,ab,kw OR 'g x e':ti,ab,kw OR 'environment':ti,ab,kw) AND ('5httlpr':ti,ab,kw OR '5-httlpr':ti,ab,kw OR 'serotonin transporter gene polymorphism':ti,ab,kw OR 'serotonin transporter gene polymorphic region':ti,ab,kw OR 'serotonin transporter linked polymorphism':ti,ab,kw OR 'serotonin transporter linked polymorphic region':ti,ab,kw OR 'serotonin transporter gene-linked polymorphic region':ti,ab,kw OR 'serotonin transporter gene-linked polymorphism':ti,ab,kw OR '5-hydroxytryptamine transporter gene-linked polymorphic region':ti,ab,kw OR '5-hydroxytryptamine transporter linked polymorphic region':ti,ab,kw OR '5-ht transporter linked polymorphic region':ti,ab,kw OR '5-ht transporter gene-linked polymorphic region':ti,ab,kw OR '5hydroxytryptamine transporter gene-linked polymorphic region':ti,ab,kw OR '5hydroxytryptamine transporter linked polymorphic region':ti,ab,kw OR '5ht transporter linked polymorphic region':ti,ab,kw OR '5ht transporter gene-linked polymorphic region':ti,ab,kw OR '5-ht t promoter polymorphism':ti,ab,kw OR '5-htt promoter polymorphism':ti,ab,kw OR 'serotonin transporter polymorphism':ti,ab,kw OR 'serotonin transporter gene promoter region polymorphism':ti,ab,kw) AND (depress*:ti,ab,kw OR 'psychological distress':ti,ab,kw OR 'mental illness*':ti,ab,kw OR 'mood disorder*':ti,ab,kw OR 'mental disorder*':ti,ab,kw OR 'mental health':ti,ab,kw OR 'psychiatric disorder*':ti,ab,kw OR 'mental disease*':ti,ab,kw OR 'psychotic disorder*':ti,ab,kw)

Scopus

TITLE-ABS-KEY ((maltreatment OR trauma* OR stress* OR "adverse" OR "life event*" OR "genetic moderation" OR "gene-environment" OR "gene-by-environment" OR "G x E" OR "environment") AND ("5httlpr" OR "5-httlpr" OR "serotonin transporter gene polymorphism" OR "serotonin transporter gene polymorphic region" OR "serotonin transporter linked polymorphism" OR "serotonin transporter linked polymorphic region" OR "serotonin transporter gene-linked polymorphic region" OR "serotonin transporter gene-linked polymorphism" OR "5-Hydroxytryptamine transporter gene-linked polymorphic region" OR "5-Hydroxytryptamine transporter linked polymorphic region" OR "5-HT transporter linked polymorphic region" OR "5-HT transporter gene-linked polymorphic region" OR "5Hydroxytryptamine transporter gene-linked polymorphic region" OR "5Hydroxytryptamine transporter linked polymorphic region" OR "5HT transporter linked polymorphic region" OR "5HT transporter gene-linked polymorphic region" OR "5-HT T promoter polymorphism" OR "5-HTT promoter polymorphism" OR "Serotonin Transporter Polymorphism" OR "Serotonin transporter gene promoter region polymorphism") AND (depress* OR "psychological distress" OR "mental illness*" OR "mood disorder*" OR "mental disorder*" OR "mental health" OR "psychiatric disorder*" OR "mental disease*" OR "psychotic disorder*"))

PubMed

(maltreatment OR trauma* OR stress* OR "adverse" OR "life event*" OR "genetic moderation" OR "gene-environment" OR "gene-by-environment" OR "G x E" OR "environment") AND ("5httlpr" OR "5-httlpr" OR "serotonin transporter gene polymorphism" OR "serotonin transporter gene polymorphic region" OR "serotonin transporter linked polymorphism" OR "serotonin transporter linked polymorphic region" OR "serotonin transporter gene-linked polymorphic region" OR "serotonin transporter gene-linked polymorphism" OR "5-Hydroxytryptamine transporter gene-linked polymorphic region" OR "5-Hydroxytryptamine transporter linked polymorphic region" OR "5-HT transporter linked polymorphic region" OR "5-HT transporter gene-linked polymorphic region" OR "5Hydroxytryptamine transporter gene-linked polymorphic region" OR "5Hydroxytryptamine

transporter linked polymorphic region" OR "5HT transporter linked polymorphic region" OR "5HT transporter gene-linked polymorphic region" OR “5-HT T promoter polymorphism” OR “5-HTT promoter polymorphism” OR “Serotonin Transporter Polymorphism” OR “Serotonin transporter gene promoter region polymorphism”) AND (depress* OR "psychological distress" OR "mental illness*" OR "mood disorder*" OR "mental disorder*" OR "mental health" OR "psychiatric disorder*" OR "mental disease*" OR "psychotic disorder*”)

PsycoInfo

(maltreatment OR trauma* OR stress* OR "adverse" OR "life event*" OR "genetic moderation" OR "gene-environment" OR "gene-by-environment" OR "G x E" OR "environment") AND ("5httlpr" OR "5-httlpr" OR "serotonin transporter gene polymorphism" OR "serotonin transporter gene polymorphic region" OR "serotonin transporter linked polymorphism" OR "serotonin transporter linked polymorphic region" OR "serotonin transporter gene-linked polymorphic region" OR "serotonin transporter gene-linked polymorphism" OR "5-Hydroxytryptamine transporter gene-linked polymorphic region" OR "5-Hydroxytryptamine transporter linked polymorphic region" OR "5-HT transporter linked polymorphic region" OR "5-HT transporter gene-linked polymorphic region" OR "5Hydroxytryptamine transporter gene-linked polymorphic region" OR "5Hydroxytryptamine transporter linked polymorphic region" OR "5HT transporter linked polymorphic region" OR "5HT transporter gene-linked polymorphic region") AND (depress* OR "psychological distress" OR "mental illness*" OR "mood disorder*" OR "mental disorder*" OR "mental health" OR "psychiatric disorder*" OR "mental disease*")

Supplementary Table 1. Classification of diagnostic tools

Study	Tool	Classification
Caspi et al 2003	DIS	Clinician-observer scales
Chipman et al 2007	GDA-S	Clinician-observer scales
	SMFQ	Self-administered scales/questionnaires
Coventry et al 2010	SSAGA	Clinician-observer scales
	HLQ	Self-administered scales/questionnaires
Cutuli et al 2013	K-SADS	Clinician-observer scales
	CDR-S	Clinician-observer scales
	SCID	Clinician-observer scales
Eley et al 2004	SMFQ	Self-administered scales/questionnaires
Fandiño-Losada, 2013	MDI	Self-administered scales/questionnaires
Gilliespie et al, 2005	SSAGA	Clinician-observer scales
Gutierrez et al 2015	CIDI	Clinician-observer scales
Haberstick et al 2016	CES-D	Self-administered scales/questionnaires
Hankin et al 2015	K-SADS	Clinician-observer scales
Juhasz et al 2015	SCID	Clinician-observer scales
	BSI	Self-administered scales/questionnaires
Kim et al 2017	MINI	Clinician-observer scales
Kudinova et al 2015	SCID	Clinician-observer scales
Laucht et al 2009	BDI	Self-administered scales/questionnaires
Özçürümez et al 2019	CIDI	Clinician-observer scales
Power et al 2008	MINI	Clinician-observer scales
	CES-D	Self-administered scales/questionnaires
Quinn et al 2012	MINI	Clinician-observer scales
Rocha et al 2015	MINI	Clinician-observer scales
Roy et al 2011	BDI	Self-administered scales/questionnaires
Sales et al 2015	CES-D	Self-administered scales/questionnaires
Surtees et al 2006	H-LEQ	Self-administered scales/questionnaires
Wilhelm et al 2006	DIS/CIDI	Clinician-observer scales

Abbreviations

BDI	Beck Depression Inventory
BSI	Brief Symptom Inventory
CDR-S	Children's Depression Rating Scale
CES-D	Center for Epidemiologic Studies-Depression Scale
CIDI	Composite International Diagnostic Interview
DIS	Diagnostic Interviews Schedule
GDA-S	Goldberg Depression and Anxiety Scales
H-LEQ	Health and Life Experiences Questionnaire
HLQ	Health and Lifestyle Questionnaire
HRSD	Hamilton Rating Scale for Depression
K-SADS-PL	Schedule for Affective Disorders and Schizophrenia for School Age Children
MDI	Major Depressive Inventory
MINI	Mini International Neuropsychiatric Interview
SCID	Structured Clinical Interview for DSM-IV

SCL-90	90-item Symptom Checklist
SMFQ	Short Mood and Feelings Questionnaire
SSAGA	Semi-Structured Assessment for the Genetics of Alcoholism

Supplementary Methods 3. Formulas

a. Formulas used to calculate Odds Ratio (OR) and related standard confidence interval (CI_{OR})

$OR = e^{\beta}$

95% Upper $CI_{OR} = e^{(\beta + SE_{\beta} \times 1.96)}$

95% Lower $CI_{OR} = e^{(\beta - SE_{\beta} \times 1.96)}$

b. Formulas used to calculate standard error (SE_{OR})

SE_{OR} = (95% Upper CI_{OR} - 95% Lower CI_{OR}) / 3.92

Abbreviations

OR = odds ratio

CI_{OR} = confidence interval of OR

β = logistic regression coefficient

SE_{β} = standard error of β

SE_{OR} = standard error of OR

Supplementary Table 2. Studies excluded in meta-analysis reporting overlapped population

Study	Sampling	Country	n	Sex (%F)	Stressor	Depression Tool	Age of depressive assessment [mean (sd)]	Stress Tool
Ancelin, 2016	Electoral rolls between 1999 and 2001 in Montpellier	France	334	49.4%	Stressful life events	MINI/CES-D	48.3 (16.6)	Gospel Oak
Artero, 2011	Electoral rolls between 1999 and 2001 in Montpellier	France	880	62%	War events	MINI	72.5 (5.1)	Questionnaires

Supplementary Table 3. Sensitivity analysis for stress

Study	OR*	95% Lower CI	95% Upper CI
**Acute Stress**			
Caspi et al 2003a	0.74	0.55	0.99
Chipman et al 2007c	0.89	0.14	5.71
Coventry et al 2010a	1.01	1.00	1.02
Gilliespie et al 2005	1.12	0.83	1.50
Kim et al 2017	1.68	1.07	2.63
Laucht et al 2009b	1.09	0.92	1.29
Power et al 2010a	0.63	0.37	1.06
Wilhelm et al 2006a	0.63	0.20	2.01
Random Effect	1.00	0.87	1.15

**Chronic Stress**			
Chipman et al 2007b	0.97	0.88	1.08
Cutuli et al 2013b	1.76	0.55	5.62
Eley et al 2004	1.85	0.91	3.77
Fandiño-Losada, 2013a	0.82	0.43	1.56
Gutierrez et al 2015	2.02	1.22	3.34
Haberstick et al 2016a	0.99	0.79	1.24
Juhasz et al 2015b	1.02	0.87	1.20
Kudinova et al 2015	0.86	0.51	1.46
Özçürümez et al 2019	1.91	0.59	6.21
Quinn et al 2012	2.41	1.10	5.26
Rocha et al 2015	1.59	1.10	2.30
Roy et al 2011	1.12	0.09	13.74
Sales et al 2015	3.79	1.20	11.97
Surtees et al 2006c	0.90	0.73	1.11
Random Effect	1.17	1.01	1.34

**Overall Effect (I^2=57%)**	**1.08**	**0.99**	**1.17**
**Test for subgroup differences: χ^2 (df=1)=2.28, p=0.13**			

*Crude OR and Adjusted OR pooled together

Supplementary Figure 1. Time interval between the end of the stress and the assessment of depression

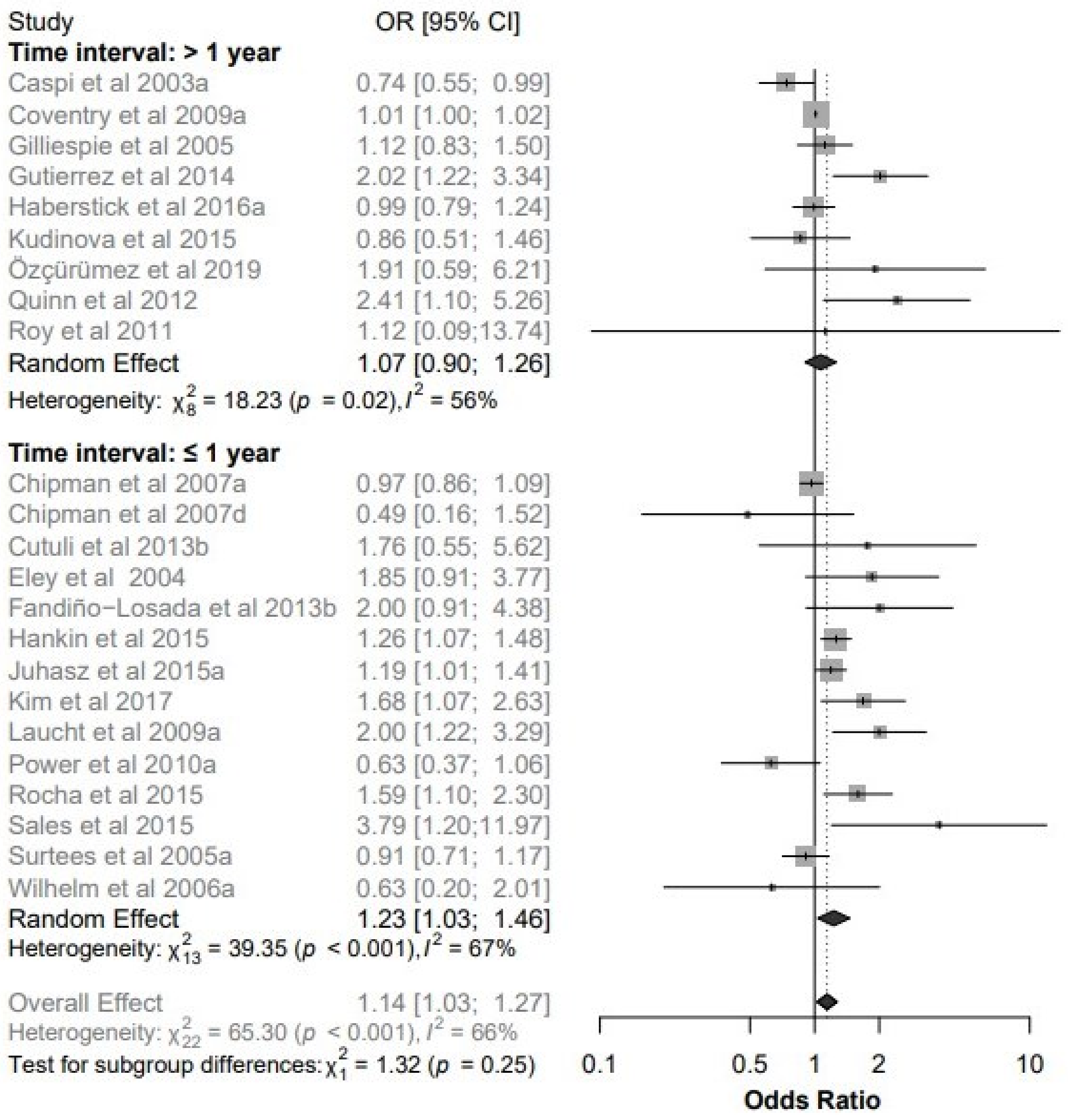

Supplementary Figure 1. Time interval between the end of the stress and the assessment of depression. Forest plot (OR and 95%IC) for 22 studies assessing the relationship between 5-HTTLPR, stress and depression stratified for time intervals (longer than one year, shorter than or equal to 1 year). The area of each square is proportional to the study weight in the analysis. The diamond represents pooled estimates from random-effects meta-analysis. Dashed line represents the overall effect. OR= Odds Ratio; CI= confidence interval.

Supplementary Table 4. Sensitivity analysis for time interval

Study	OR*	95% Lower CI	95% Upper CI
**Timespan > 1 year**			
Caspi et al 2003a	0.74	0.55	0.99
Chipman et al 2007b	0.97	0.88	1.08
Coventry et al 2010a	1.01	1.00	1.02
Cutuli et al 2013a	2.16	0.53	8.85
Fandiño-Losada, 2013a	0.82	0.43	1.56
Gilliespie et al 2005	1.12	0.83	1.50
Gutierrez et al 2015	2.02	1.22	3.34
Haberstick et al 2016a	0.99	0.79	1.24
Juhasz et al 2015b	1.02	0.87	1.20
Kudinova et al 2015	0.86	0.51	1.46
Laucht et al 2009b	1.09	0.92	1.29
Özçürümez et al 2019	1.91	0.59	6.21
Quinn et al 2012	2.41	1.10	5.26
Roy et al 2011	1.12	0.09	13.74
Surtees et al 2006b	0.96	0.84	1.09
Wilhelm et al 2006b	0.56	0.32	0.97
Random Effect	1.00	0.94	1.08
**Timespan ≤ 1 year**			
Chipman et al 2007c	0.89	0.14	5.71
Eley et al 2004	1.85	0.91	3.77
Hankin et al 2015	1.26	1.07	1.48
Kim et al 2017	1.68	1.07	2.63
Power et al 2010a	0.63	0.37	1.06
Rocha et al 2015	1.59	1.10	2.30
Sales et al 2015	3.79	1.20	11.97
Random Effect	1.37	1.02	1.84
**Overall Effect (I^2=60%)**	**1.07**	**0.98**	**1.16**
**Test for subgroup differences: χ^2 (df=1)=4.15, p=0.04**			

*Crude OR and Adjusted OR pooled together

Supplementary Table 5. Sensitivity analysis for time interval (only chronic stress)

Study	OR*	95% Lower CI	95% Upper CI
**Timespan > 1 year**			
Chipman et al 2007b	0.97	0.88	1.08
Cutuli et al 2013a	2.16	0.53	8.85
Fandiño-Losada et al 2013a	0.82	0.43	1.56
Gutierrez et al 2015	2.02	1.22	3.34
Haberstick et al 2016	0.99	0.79	1.24
Juhasz et al 2015b	1.02	0.87	1.20
Kudinova et al 2015	0.86	0.51	1.46
Özçürümez et al 2019	1.91	0.59	6.21
Quinn et al 2012	2.41	1.10	5.26
Roy et al 2011	1.12	0.09	13.74
Surtees et al 2006c	0.90	0.73	1.11
Random Effect	1.04	0.91	1.18
**Timespan ≤ 1 year**			
Chipman et al 2007d	0.49	0.16	1.52
Eley et al 2004	1.85	0.91	3.77
Hankin et al 2015	1.26	1.07	1.48
Laucht et al 2009a	2.00	1.22	3.29
Rocha et al 2015	1.59	1.10	2.30
Sales et al 2015	3.79	1.20	11.97
Random Effect	1.53	1.14	2.07
**Overall Effect (I^2=63%)**	**1.20**	**1.04**	**1.39**
**Test for subgroup differences: χ^2 (df=1)=5.49, p=0.02**			

*Crude OR and Adjusted OR pooled together

Supplementary Figure 2. Time interval between the end of the stress and the assessment of depression only in chronic stress studies

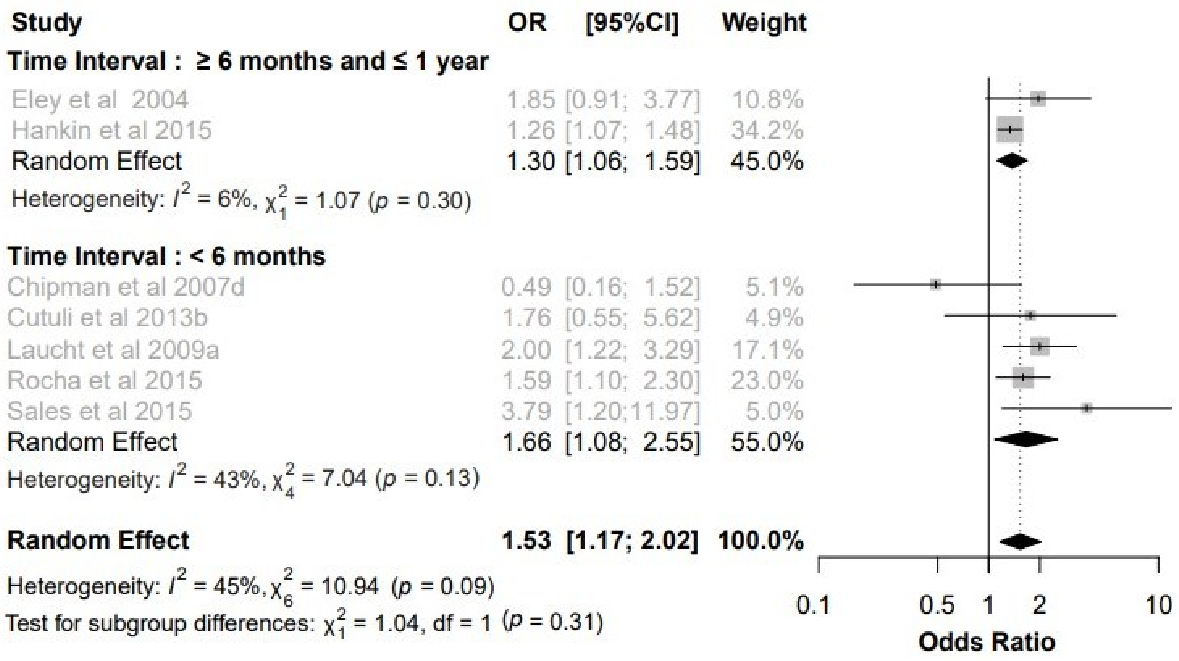

Supplementary Figure 2. Time interval between the end of the stress and the assessment of depression only in chronic stress studies. Forest plot (OR and 95%IC) for 16 studies assessing the relationship between 5- HTTLPR, stress and depression stratified for two time intervals (between 1 year and 6 months and shorter than 6 months). The area of each square is proportional to the study weight in the analysis. The diamond represents pooled estimates from random-effects meta-analysis. Dashed line represents the overall effect. OR= Odds Ratio; CI= confidence interval.

Supplementary Figure 3. Diagnostic tool

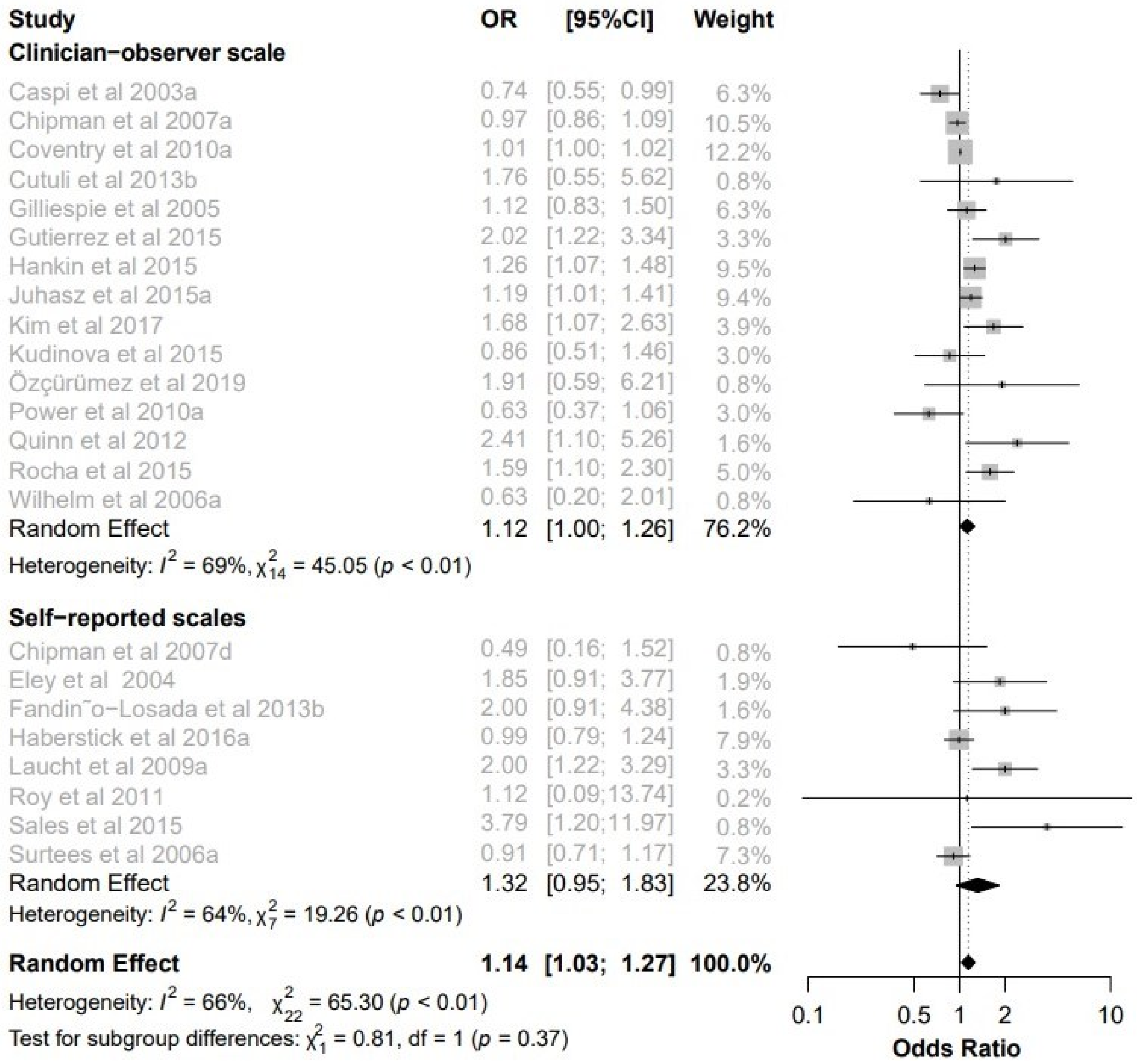

Supplementary Figure 3. Diagnostic tool. Forest plot (OR and 95%IC) for 22 studies assessing the relationship between 5-HTTLPR, stress and depression stratified by diagnostic tool (clinician-observer scales vs self-reported scales). The area of each square is proportional to the study weight in the analysis. The diamond represents pooled estimates from random-effects meta-analysis. Dashed line represents the overall effect. OR= Odds Ratio; CI= confidence interval.

Supplementary Table 6. Sensitivity analysis for diagnostic tool

Study	OR*	95% Lower CI	95% Upper CI
**Clinician-observer scales**			
Caspi et al 2003a	0.74	0.55	0.99
Chipman et al 2007a	0.97	0.86	1.09
Cutuli et al 2013b	1.76	0.55	5.62
Gilliespie et al 2005	1.12	0.83	1.50
Gutierrez et al 2015	2.02	1.22	3.34
Hankin et al 2015	1.26	1.07	1.48
Kim et al 2017	1.68	1.07	2.63
Kudinova et al 2015	0.86	0.51	1.46
Özçürümezet al 2019	1.91	0.59	6.21
Power et al 2010a	0.63	0.37	1.06
Quinn et al 2012	2.41	1.10	5.26
Rocha et al 2015	1.59	1.10	2.30
Wilhelm et al 2006a	0.63	0.20	2.01
Random Effect	1.22	1.01	1.47
**Self-reported scales**			
Chipman et al 2007c	0.89	0.14	5.71
Eley et al 2004	1.85	0.91	3.77
Coventry et al 2010b	1.03	1.00	1.02
Fandiño-Losada et al 2013b	2.00	0.91	4.38
Haberstick et al 2016	0.99	0.79	1.24
Laucht et al 2009a	2.00	1.22	3.29
Roy et al 2011	1.12	0.09	13.74
Sales et al 2015	3.79	1.20	11.97
Juhasz et al 2015c	1.08	1.00	1.16
Surtees et al 2006a	0.91	0.71	1.17
Random Effect	1.13	0.96	1.32
**Overall Effect (I^2=61%)**	**1.17**	**1.04**	**1.31**
**Test for subgroup differences: χ^2 (df=1)=0.39, p=0.53**			

*Crude OR and Adjusted OR pooled together

Supplementary Table 7. JBI critical appraisal tool

a. Longitudinal studies

Study	Q1	Q2	Q3	Q4	Q5	Q6	Q7	Q8	Q9	Q10	Q11	%Yes	Risk
Caspi, 2003	✓	✓	✓	✓	✓	?	✓	✓	×	#	✓	73%	Low
Chipman, 2007 a	✓	✓	✓	×	#	?	✓	✓	×	#	✓	55%	Moderate
Coventry, 2010	✓	✓	✓	×	#	✓	✓	✓	×	#	✓	64%	Moderate
Cutuli, 2013	✓	✓	✓	×	#	?	✓	✓	✓	×	✓	64%	Moderate
Fandiño-Losada et al 2013	✓	✓	✓	✓	✓	?	✓	✓	×	#	✓	73%	Low
Gutierrez, 2015	✓	✓	✓	✓	✓	✓	✓	✓	✓	×	✓	91%	Low
Haberstick, 2016	✓	✓	✓	×	#	?	✓	✓	×	#	✓	55%	Moderate
Hankin, 2015	✓	✓	✓	✓	✓	✓	✓	✓	✓	×	✓	91%	Low
Kim, 2017	✓	✓	✓	✓	✓	?	✓	✓	✓	×	✓	82%	Low
Power, 2010	✓	✓	✓	×	#	✓	✓	✓	×	#	✓	64%	Moderate
Rocha, 2015	✓	✓	✓	✓	✓	✓	✓	✓	✓	✓	✓	100%	Low
Wilhelm, 2006	✓	✓	✓	✓	✓	#	✓	✓	✓	×	✓	82%	Low

b. Cross-sectional studies

Study	Q1	Q2	Q3	Q4	Q5	Q6	Q7	Q8	%Yes	Risk
Chipman, 2007 b	✓	✓	✓	✓	×	#	✓	✓	75%	Low
Eley, 2004	×	×	✓	✓	×	#	✓	✓	50%	Moderate
Gilliespie, 2005	×	✓	✓	✓	×	#	✓	✓	63%	Moderate
Juhasz ,2015	×	✓	✓	✓	✓	✓	✓	✓	88%	Low
Laucht, 2009	✓	✓	✓	✓	✓	✓	✓	✓	100%	Low
Roy, 2011	✓	✓	✓	✓	×	#	✓	✓	75%	Low
Sales, 2015	✓	✓	✓	✓	✓	✓	✓	✓	100%	Low
Surtees, 2006	✓	✓	✓	✓	✓	✓	✓	✓	100%	Low

c. Case-control studies

Study	Q1	Q2	Q3	Q4	Q5	Q6	Q7	Q8	Q9	Q10	%Yes	Risk
Kudinova, 2015	?	?	?	✓	✓	×	#	✓	✓	✓	50%	Moderate
Özçürümez, 2019	✓	✓	✓	✓	✓	✓	✓	✓	✓	✓	100%	Low
Quinn, 2012	✓	✓	✓	✓	✓	✓	✓	✓	✓	✓	100%	Low

Notes. Qs indicate questions based on the JBI risk assessment

The risk of bias was ranked as high when the study reached up to 49% of “yes” scores, moderate when the study reached from 50 to 69% of “yes” scores, and low when the study reached more than 70% of “yes” scores. ‘✓’ indicates yes, ‘×’ indicates no and ‘?’ indicates unclear, ‘#’ indicates not applicable

Supplementary Figure 4. Funnel Plot

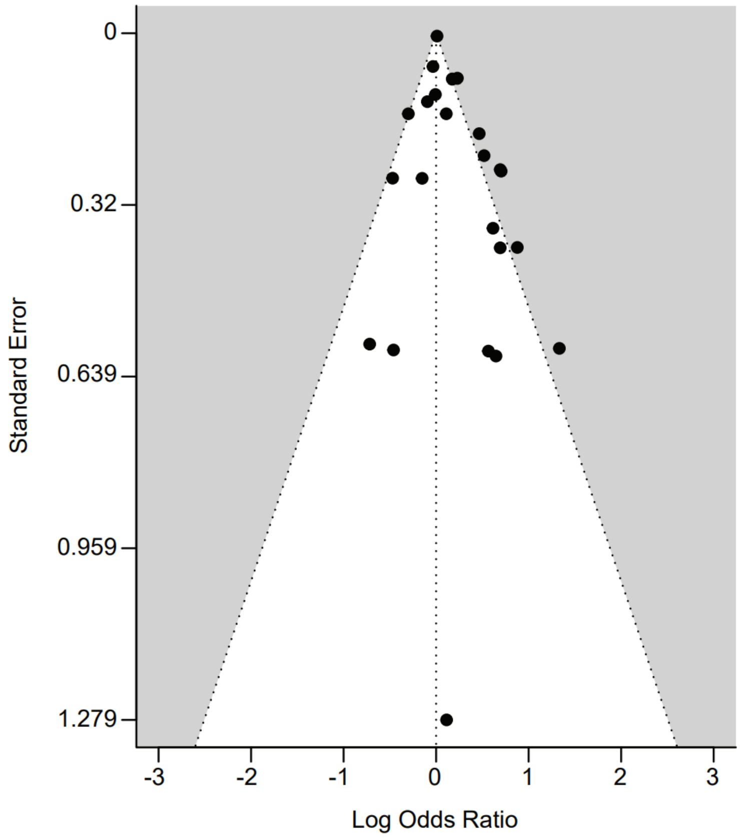

Supplement: Supplementary file 1 — Supplementary Material [file 41398_2022_2035_MOESM1_ESM.pdf]
